# Molecular identification, phylogenetic analysis and histopathological study of pathogenic free-living amoebae isolated from discus fish (*Symphysodon aequifasciatus*) in Iran: 2020–2022

**DOI:** 10.1186/s12917-024-03902-6

**Published:** 2024-02-12

**Authors:** Hooman Rahmati-Holasoo, Maryam Niyyati, Marziye Fatemi, Fatemeh Mahdavi Abhari, Sara Shokrpoor, Alireza Nassiri, Amin Marandi

**Affiliations:** 1 Department of Aquatic Animal Health, Faculty of Veterinary Medicine, University of Tehran, Tehran, Iran; 2 Department of Medical Parasitology and Mycology, School of Medicine, Shahid Beheshti University of Medical Sciences, Tehran, Iran; 3 Student Research Committee, School of Medicine, Shahid Beheshti University of Medical Sciences, Tehran, Iran; 4 Department of Pathology, Faculty of Veterinary Medicine, University of Tehran, Tehran, Iran

**Keywords:** *Acanthamoeba*, Discus fish, Epithelial hyperplasia, Iran, Nodular gill disease

## Abstract

Free-living amoebae (FLA) are capable of inhabiting diverse reservoirs independently, without relying on a host organism, hence their designation as “free-living”. The majority of amoebae that infect freshwater or marine fish are amphizoic, or free-living forms that may colonize fish under particular circumstances. *Symphysodon aequifasciatus*, commonly referred to as the discus, is widely recognized as a popular ornamental fish species. The primary objective of the present study was to determine the presence of pathogenic free-living amoebae (FLA) in samples of discus fish. Fish exhibiting clinical signs, sourced from various fish farms, were transferred to the ornamental fish clinic. The skin, gills, and intestinal mucosa of the fish were collected and subjected to culturing on plates containing a 1% non-nutrient agar medium. The detection of FLA was conducted through morphological, histopathological and molecular methods. The construction of the phylogenetic tree for Acanthamoeba genotypes was achieved using the maximum likelihood approach. The molecular sequence analysis revealed that all cultures that tested positive for FLA were T4 genotype of Acanthamoeba and *Acanthamoeba sp.* The examination of gill samples using histopathological methods demonstrated the presence of lamellar epithelial hyperplasia, significant fusion of secondary lamellae, and infiltration of inflammatory cells. A multitude of cysts, varying in shape from circular to elliptical, were observed within the gills. The occurrence of interlamellar vesicles and amoeboid organisms could be observed within the epithelial tissue of the gills. In the current study, presence of the Acanthamoeba T4 genotype on the skin and gills of discus fish exhibiting signs of illness in freshwater ornamental fish farms was identified. This observation suggests the potential of a transmission of amoebic infection from ornamental fish to humans, thereby highlighting the need for further investigation into this infection among ornamental fish maintained as pets, as well as individuals who interact with them and their environment.

## Introduction

Aquaculture is expanding rapidly. The most key determinants of the aquaculture industry are edible and aquarium fish cultivation [1, 2]. Ornamental fish culture has transformed into one of aquaculture’s most significant aspects, and it is acknowledged as one of the most lucrative sectors in Iran and many other countries [3–7]. The main production of ornamental fish is in the south eastern Asia [8]. More than 150 freshwater fish species are currently farmed as ornamental fish in Iran [9]. Discus fish (*Symphysodon aequifasciatus*) is a popular freshwater perciform ornamental fish belonging to the Cichlidae family [10, 11]. Breeding of discus fish has progressed significantly in Iran over the last few years [9].

There is a claimed increase in the global prevalence and incidence of emerging diseases observed in many organisms. Parasitic diseases are prevalent among a significant proportion of fish in both wild and cultured populations, hence raising substantial concerns over the welfare of ornamental fish [12]. Amoebae are a large polyphyletic group of protozoan eukaryotes distinguished by their ability to shape-change and amoeboid movement, primarily through the development of pseudopods [13–15], and consist of members from many supergroups, including *Amoebozoa*, *Alveolata, Excavata*, *Heterokonta*, *Opistokonta*, and *Rhizaria* [16, 17]. These ubiquitous organisms are widespread in a variety of marine and freshwater habitats with various sizes and hosts that range from invertebrates to humans [13, 17]. The majority of amoeba-fish relationships are divided into four categories: ectoparasites, ectocommensals, endoparasites, and endocommensals, though the distinctions are not always evident. While some, particularly commensals, do not damage fish, they can also act as parasites in a variety of situations [13]. Amoebae can feed on bacterial biofilms [18–21] as well as fish gills and skin. Furthermore, the gills and skin mucus can nourish the amoebae [13].

The majority of amoebae that infect freshwater or marine fish are amphizoic, or free-living forms that may colonize fish under particular circumstances. They would be extremely offensive within the host and cause severe mortalities. However, the amoeba genera isolated or discovered on the gills of Atlantic salmon (*Salmo salar*) are likely the most studied. Amoeba-caused gill infections are common in a wide range of marine and freshwater fish species [19]. Although AGD (amoebic gill disease) is used to represent amoebic infection in marine fish, NGD (nodular gill disease), typically refers to amoebic infection in freshwater fish species and is thought to be caused by multiple amoeba species (*Acanthamoeba*, *Hartmannella*, *Naegleria*, *Protacanthamoeba Vannella* and *Roghostoma*) taking part in various stages of disease progression [13, 22, 23]. Nodular gill disease has been reported in a variety of fish species, including coho salmon (*Oncorhynchus kisutch*) [24], goldfish (*Carassius auratus*) [25], rainbow trout (*Oncorhynchus mykiss*) [22, 26–29], arctic charr (*Salvelinus alpinus*) [30], pallid sturgeon (*Scaphirhynchus albus*) [31], chinook salmon (*Oncorhynchus tshawytscha*) [24, 32], and brown trout (*Salmo trutta*) [33].

Despite the potential for a wide geographic distribution of free-living pathogenic amoebae, little is recognized about their ecological role in surface waters, as well as their potential role in the etiology of fish disease. Based on our information, this is the first research in Iran to detect *Acanthamoeba* genotype T4 skin and gill infections using clinical, histopathological, and molecular analysis of discus fish (*S. aequifasciatus*).

## Materials and methods

### Fish origin and laboratory examinations

**Fig. 1:**
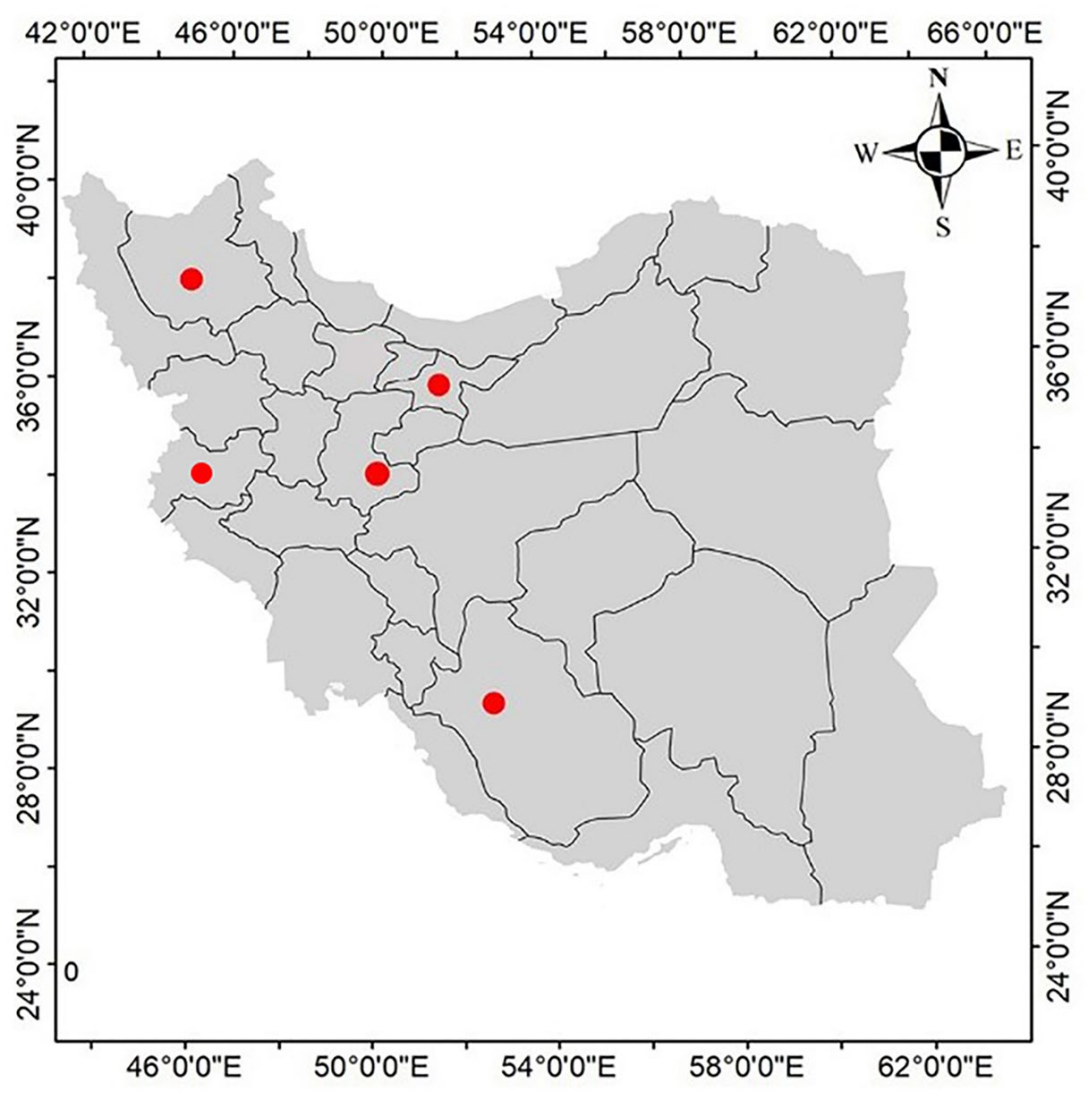
Sampling locations of freshwater ornamental fish farms (●) in Iran (2019–2022)

**Fig. 2:**
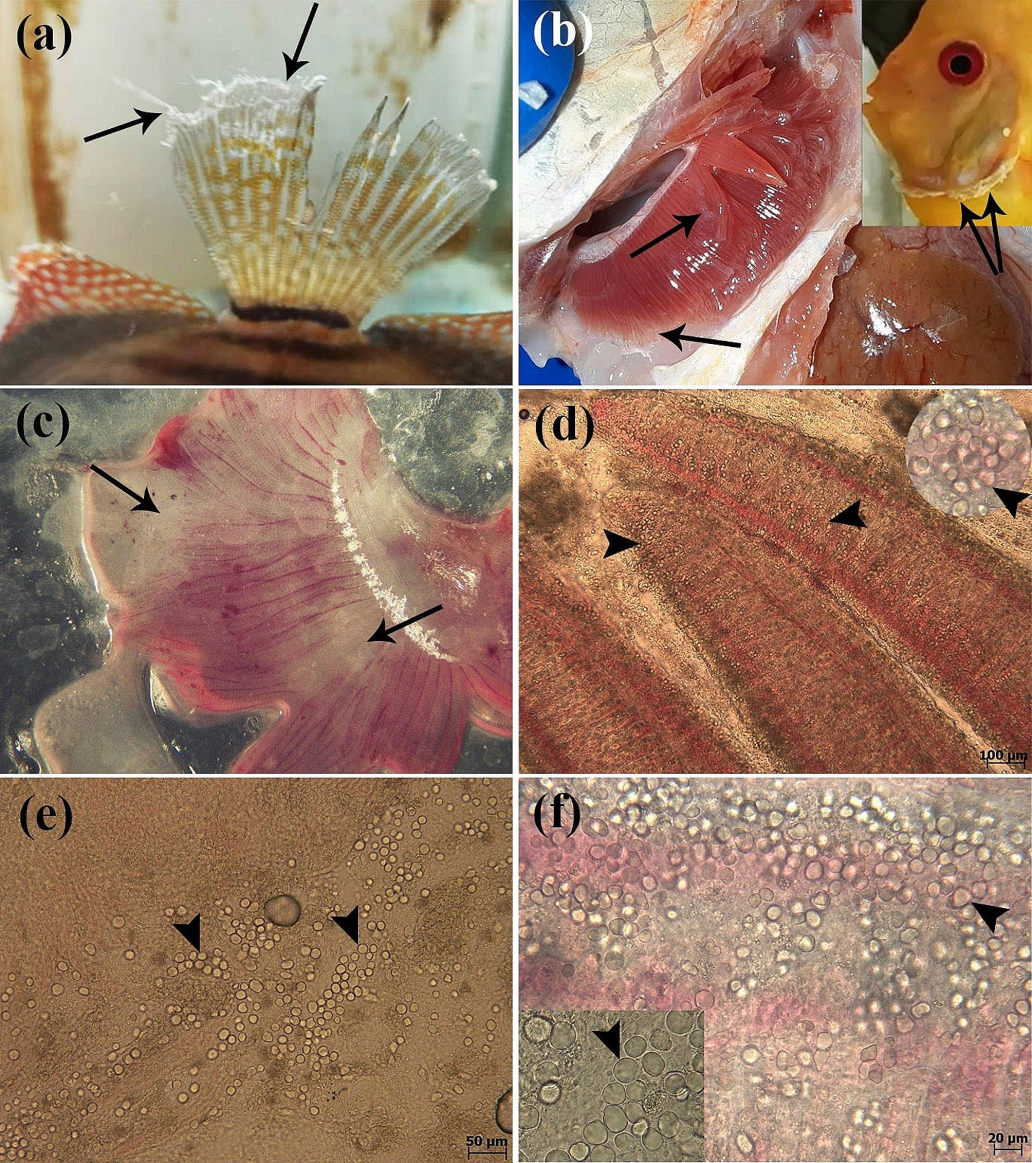
Infected discus fish (*S. aequifasciatus*) with amoebic organisms. (**a**, **b**, **c**) Mucus hypersecretion (arrows) on the caudal fin, gills and branchiostegal membrane (inset) of discus fish (*S. aequifasciatus*) are seen. (**d**, **e**, **f**). A large number of amoebic organisms (arrowheads) are seen in wet smears of skin (**e**) and gills (**d**&**f**)

During the period from February 2020 to February 2022, a complex of clinical signs in cultured freshwater ornamental discus fish (*S. aequifasciatus*) was observed in several freshwater ornamental fish farms (farming only discus). These farms were located in different geographical regions of Iran (Fig. 1). A complex of clinical signs including lethargy, anorexia, increased opercular ventilation rate (rapid opercular movements), and mucus hypersecretion on the gills and skin of discus fish (*S. aequifasciatus*) (Fig. 2a, b &c) were accompanied by significant mortalities (up to 70% monthly), were reported. Fish were fed a diet primarily composed of raw beef heart mix (3% of body weight per day). Furthermore, the temperature of the aquarium water fluctuated between 29 and 31 °C, and the pH ranged from 6.8 to 7.0. 40 (8 fish per each farm including 2 apparently normal fish, 6 fish with clinical signs) discus fish (*S. aequifasciatus*) (length of 5-17.5 cm) were packed in water-filled polyethylene bags, oxygenated, and referred to the ornamental fish clinic, Faculty of Veterinary Medicine, University of Tehran (Tehran, Iran). The duration of fish transportation was different based on the distance from the farms to the clinic. The minimum time was 1 h for the nearest farm and the maximum time for the farthest farm was 20 h. 95% of fish were still alive when they arrived at the clinic. Then wet smears of skin, fins, and gills were prepared and examined under a light microscopy (E600, Nikon). Gills were also investigated using a trinocular stereomicroscope (SZ60, Olympus) (Fig. 2c). Subsequently, with the consent of owners, the alive fish were euthanized by overdose of PI222 (10 ml/10 lit) (Eugenol, Carvacrol, and Eugenol acetate are the main active ingredients in PI222) (Pars Imen Daru, Iran), and necropsy was performed under the sterile conditions. Following the examination of internal organs by light microscopy (E600, Nikon), aerobic and anaerobic bacterial cultures from the kidney, spleen, and liver were streaked on blood agar (BA), MacConkey agar (MAC), and Trypticase Soy Agar (TSA) and incubated at 25 °C for 96 h. In addition, fungal cultures from the external and internal organs were streaked on Sabouraud dextrose agar (SDA) and incubated at 28 °C for 240 h.

Subsequently, an amoeba culture was performed on 1% non-nutrient agar medium using skin, gills, and intestinal mucosa. Parafilm was used to seal culture plates, preventing evaporation and contamination of the cultures by outside organisms, and culture plates were then incubated at 25–30 °C, and monitored for presence of amoeba trophozoite/cyst daily until 60 days. The collected samples were studied under an optical microscope with a magnification of ×100, and amoebas were identified considering morphological characteristics of cysts and trophozoites according to Niyyati and Rezaeian [34]. Morphology of the cultured amoebae was determined using Page key [35]. Continuous and successive cultivation was used to achieve pure amoeba growth in plates free of fungi and bacteria. The lack of fungi and bacteria was achieved by subculturing and cloning a few amoebae from first plate. The amoeba-containing precipitate was kept in a 20 °C freezer until DNA extraction. For the rest of fish, gradual increase of water salinity to 5 g/l (7 days) as well as usage of formalin bath (1–2 ml for 24 h, then 90% of water was changed and the treatment was repeated for 5 times), metronidazole (5–7 mg/l for 24 h, then 90% of water was changed and the treatment was repeated for 5 times, chloramine-T (0.01 g/l for 8 h, every other day), and antibiotic therapy (tetracycline cephalexin, and oxytetracycline 5–7 mg/l for 24 h, 5 days) had been performed.

### Histopathological study

For histological evaluations, skin, gills, and internal organs (e.g., spleen, liver, heart and gastrointestinal tract) were dissected and fixed in 10% neutral buffered formalin (NBF). The fixed samples were dissected, dehydrated in a series of ethanol, and finally embedded in paraffin using a paraffin tissue processor and paraffin dispenser. Subsequently, several 4 μm sections were cut from each block and stained with hematoxylin and eosin (H&E). Furthermore, Periodic acid-Schiff (PAS) and Masson’s trichrome (MT) were used to stain some of the sections. The sections were examined by light microscopy (E600, Nikon), and representative images were taken by a GT12 microscope camera (Tucsen, Mosaic 2.4 software). Axiovision version 4.8 was used to apply the image scale bars, and Adobe Photoshop CC 2023 was used to process the images.

### DNA extraction and polymerase chain reaction (PCR)

The trophozoites and cysts were extracted with phosphate buffered saline (PBS) using a cell scraper. DNA was extracted directly from skin and gill tissue homogenates using the DNA extraction kit according to the manufacturer’s instructions (CinnaGen, Iran) and stored at -20 ◦C until further analysis. A PCR assay was performed for three different genera of free-living amoebae (FLA) including *Acanthamoeba*, *Vahlkampfiids*, and *Vermamoeba* using specific primers (JDP1 & 2, ITS1 & 2, and Hv1227F & Hv1728R, respectively) (Table 1) [36–38]. Negative control was performed using sterile distilled water in the master mix instead of DNA. The polymerase chain reaction was performed in a total volume of 25 µl, which included 12.5 µl 2x Master Mix, 1 µl of each 10µM primer, 2 µl of extracted DNA, and 8.5 µl of distilled water. The cycling conditions included an initial denaturation at 94 °C for 5 min was followed by 35 cycles at 94 °C for 45 s, 56 °C for 45 s, 72 °C for 45 s, and 72 °C for 7 min as the final extension. The amplification products (5 µl) were electrophoresized on a 1.2% agarose gel, stained with 1 µg/ml ethidium bromide (CinnaGen, Iran), and screened with a UV-Trans-illuminator.

**Table 1:** Primer sets for three genera of free-living amoebae were used in the present study

Free-living amoeba	Primer’s name	Target gene	Primers 5´ → 3´	Amplicon size (bp)	Reference
*Acanthamoeba* spp.	JDP	SSU rRNA	*GGCCCAGATCGTTTACCGTGAA* *TCTCACAAGCTGCTAGGGAGTCA*	~500	[36]
*Vahlkampfiids*	ITS	Internal Transcribed Spacer (ITS)	*GAACCTGCGTAGGGATCATTT* *TTTCTTTTCCTCCCCTTATTA*	~400–700	[37]
*Vermamoeba vermiformis* (formerly named *Hartmannella vermiformis*)	Hv	18 S ribosomal RNA	*TTACGAGGTCAGGACACTGT* *GACCATCCGGAGTTCTCG*	~500	[38]

### Sequencing, homology, and phylogenetic analysis

The amplified PCR products that were positive were sequenced and Chromas 2.6.5 software was used for chromatogram analysis to obtain the consensus sequences. Homology analysis of sequence results was performed by the Basic Local Alignment Search Tool (BLAST) and compared with the GenBank sequence database. Multiple sequences were aligned using ClustalW (BioEdit version 7.0) and phylogenetic analysis was performed by 1000-fold bootstrap replications accuracy using MEGA software version 7.0.

## Results

### Clinical and laboratory examinations

Following the initial macroscopic examination (on the farm and in the clinic), lethargy, inappetence, body discoloration, and mucus overproduction on the gills and skin of discus fish (*S. aequifasciatus*) were observed. No bacterial growth appeared on blood agar (BA), MacConkey agar (MAC), and Trypticase Soy Agar (TSA), as was no fungal growth on Sabouraud dextrose agar (SDA). Furthermore, no fungi were found during a microscopic inspection of the external and internal organs. Wet smears of skin and gills revealed the presence of a large number of amoebic-like organisms in (Fig. 2d, e & f). No metazoan parasites were detected in gills and internal organs. Various treatment protocols were followed, including the use of a salt bath as well as formalin, metronidazole, and various antibiotics (such as tetracycline, cephalexin, and oxytetracycline), all of which were ineffective or had little effect. Only the use of chloramine-T (0.01 g/l) for 8 h every other day prevented the mortalities and reduced infection intensity, though the infestation was not completely eliminated.

### Histopathological analysis

**Fig. 3:**
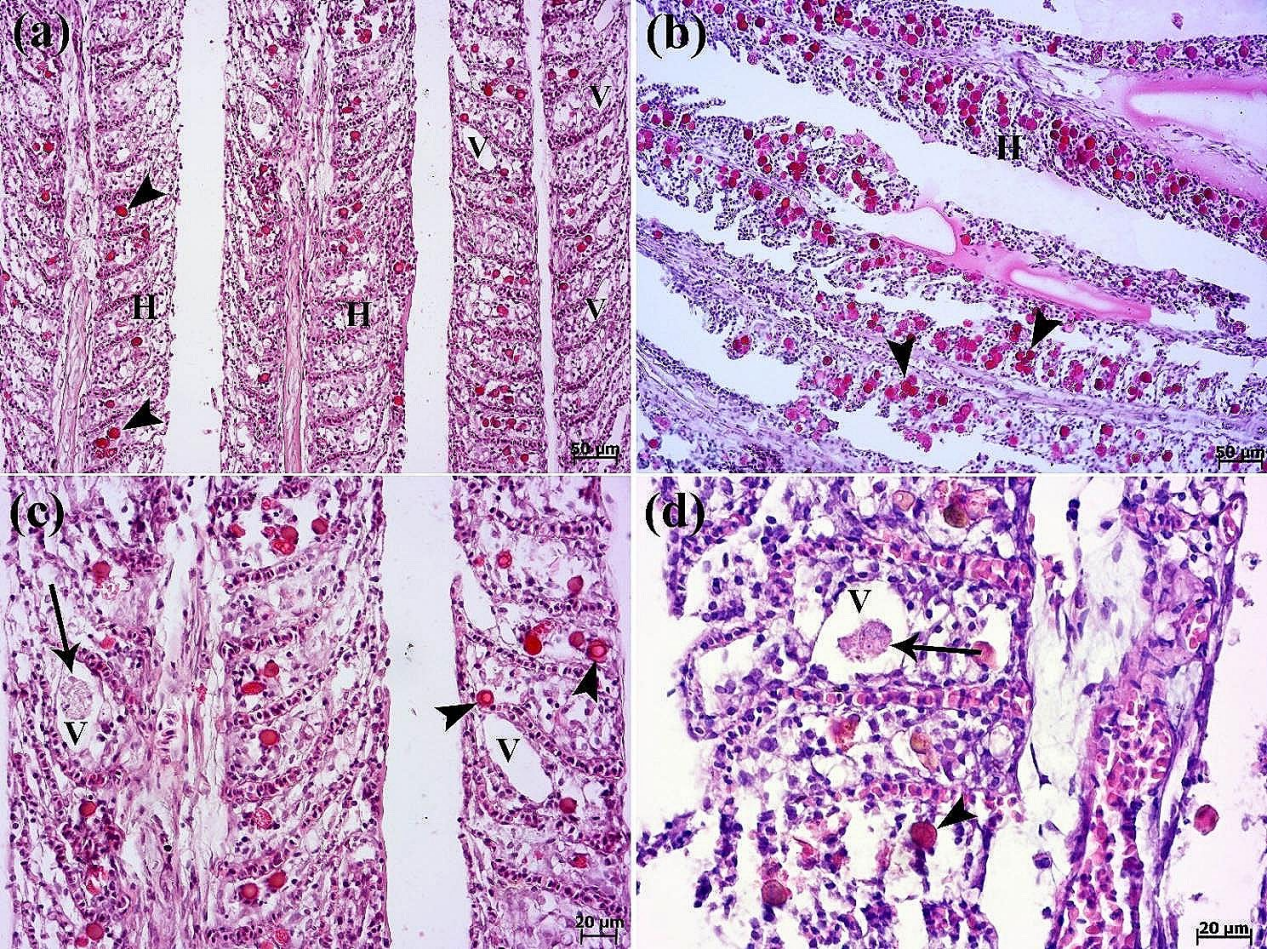
Histopathological findings of gills. (**a**-**b**) Mild to severe infestation of secondary lamellae of gills with cysts of *Acanthamoeba* sp. (**a**) Lamellar epithelial hyperplasia (H) and interlamellar vesicles formation (V) and cysts (arrowheads). (**b**) Numerous cysts (arrowheads) of *Acanthamoeba* sp. and lamellar epithelial hyperplasia (H) are seen. (**c**-**d**) Higher magnifications show trophozoite (arrow) and cysts (arrowheads) of amoebae trapped inside the hyperplastic epithelial tissue. Interlamellar vesicles formation (V) are seen (H&E).

**Fig. 4:**
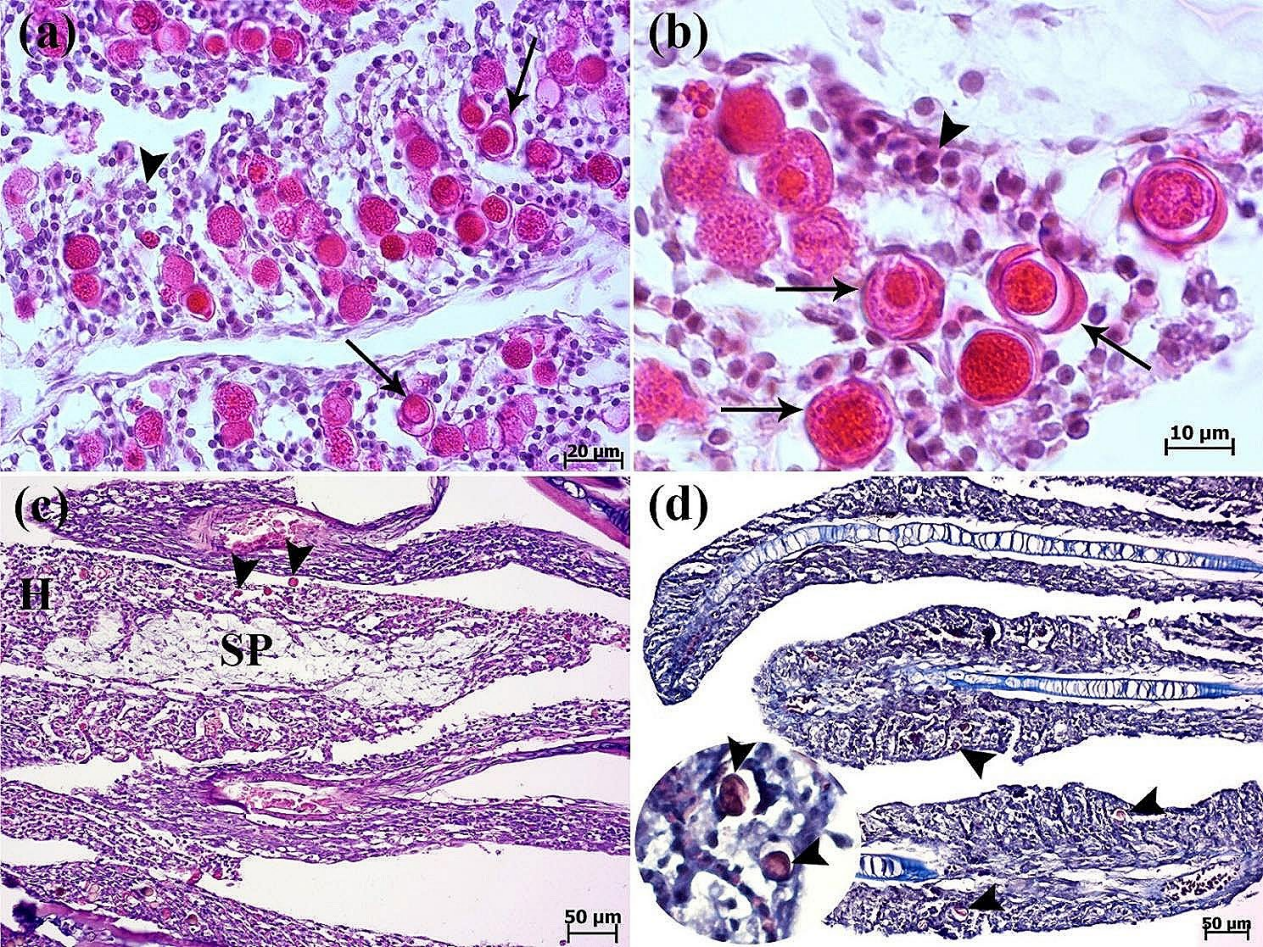
Histopathological findings of gills. (**a**) Inflammatory cells infiltration (arrowhead) and presence of numerus cysts of *Acanthamoeba* sp. (arrows) inside the hyperplastic epithelial tissue of gills. (**b**) Higher magnification shows cysts with double-layered walls and central nucleus (arrows). Inflammatory cells infiltration (arrowhead). (**c**) Gill filament characterized by spongiosis (SP) of hyperplastic (H) epithelium. Cysts of *Acanthamoeba* sp. (arrowheads) (H&E). (**d**) The Masson’s trichrome staining highlights the cysts of *Acanthamoeba* sp. (arrowheads)

**Fig. 5:**
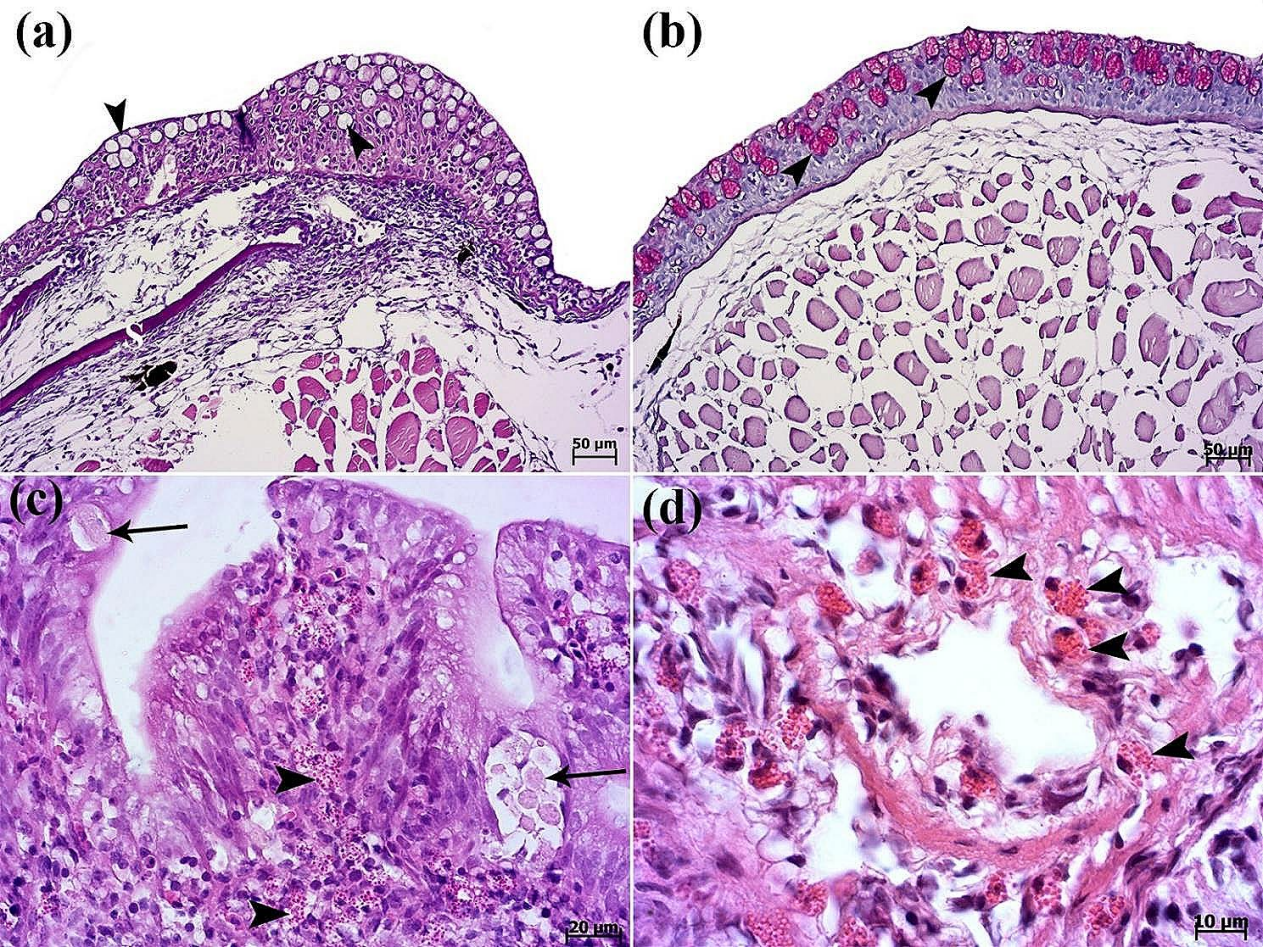
Histopathological findings of skin and intestinal tissue. (**a**) Hyperplasia of goblet cells (arrowheads) in the epidermis (H&E). Scale (S). (**b**) Periodic acid-Schiff (PAS) reaction showing the positive-stained goblet cells (arrowheads). (**c**) *Acanthamoeba* sp. trophozoites (arrows) with foamy appearance in the intestine of discus fish. (**d**) Eosinophilic granular cells (arrowheads) (H&E).

Histopathological evaluation of gills revealed lamellar epithelial hyperplasia, extensive fusion of secondary lamellae (Fig. 3a), and inflammatory cells infiltration (specially lymphocyte cells) (Figs. 3a and 4a). Also, development of round to oval interlamellar vesicles (interlamellar spaces or cavities) was observed. Amoeboid organisms were found embedded in the epithelia of the gills (Figs. 3a-d and 4a, amp and b). Ovoid to round amoebae consistent with trophozoites, measuring 20 to 25 μm in size were seen inside the hyperplastic epithelial tissue (Fig. 3c & d). In gills of all fish, amoebic cysts were seen round to oval. The amoebic cysts were thin to thick-walled structures with a diameter of 12 to 16 μm and round-like nucleus (Fig. 4a & b). Spongiosis of hyperplastic gill epithelium was observed in some cases (Fig. 4c). Cysts of *Acanthamoeba* sp. were also stained with Masson’s trichrome (Fig. 4d). Increasing number of epidermal goblet cells were obvious in compare with the normal discus fish (archive of fish histopathology laboratory, Faculty of Veterinary Medicine, University of Tehran). Periodic acid-Schiff (PAS) reaction showed the positive-stained goblet cells (Fig. 5a & b). Parasitic and eosinophilic enteritis due to presence of trophozoites and eosinophilic granular cells were identified. Trophozoites of *Acanthamoeba* sp. with foamy cytoplasm were observed in some cases due to the presence of prominent vacuoles in the intestine (Fig. 5c & d). On the contrary, other internal organs were normal.

### PCR detection

Specific amplicons with an expected size of 500 bp were detected by PCR assay via JDP1 & 2 primer set in the homogenates of skin and gills collected from the moribund fish based on PCR amplification of *Acanthamoeba-SSU rRNA* gene results from infected discus fish. There was no amplicon in the negative control.

### Sequences analysis

Acanthamoebiasis was also confirmed by PCR amplification sequencing, as a GenBank BLAST search on the sequence revealed the highest similarity to previously published sequences of the *Acanthamoeba-SSU rRNA* gene. As a result, the parasite’s nucleotide sequences have been added to the GenBank (NCBI) database as OP910247 (AF2), OP910249 (AF7), OP910251 (AF22), OP910248 (AF3), and OP910250 (AF8). Alignment and phylogenetic analysis of the OP910247 (AF2), OP910249 (AF7), OP910251 (AF22), OP910248 (AF3), and OP910250 (AF8) classified the parasites into the genotype T4 (Table 2).

**Table 2:** Data regarding the source and the isolated genotype of *Acanthamoeba* from Discus fish samples in Iran

Code	Source	Genus	Species (genotype)	Similar Accession number	Identity (%)/Query coverage (%)	Accession number
AF2	Skin	*Acanthamoeba*	T4	LC177666.1	100/100	OP910247
AF3	Skin	*Acanthamoeba*	T4	KT892910.1	100/100	OP910248
AF7	Gill	*Acanthamoeba*	Sp.	MN153023.1	99.51/100	OP910249
AF8	Gill	*Acanthamoeba*	T4	KT892916.1	100/99	OP910250
AF22	Skin	*Acanthamoeba*	T4	KR074228.1	99.5/100	OP910251

**Fig. 6:**
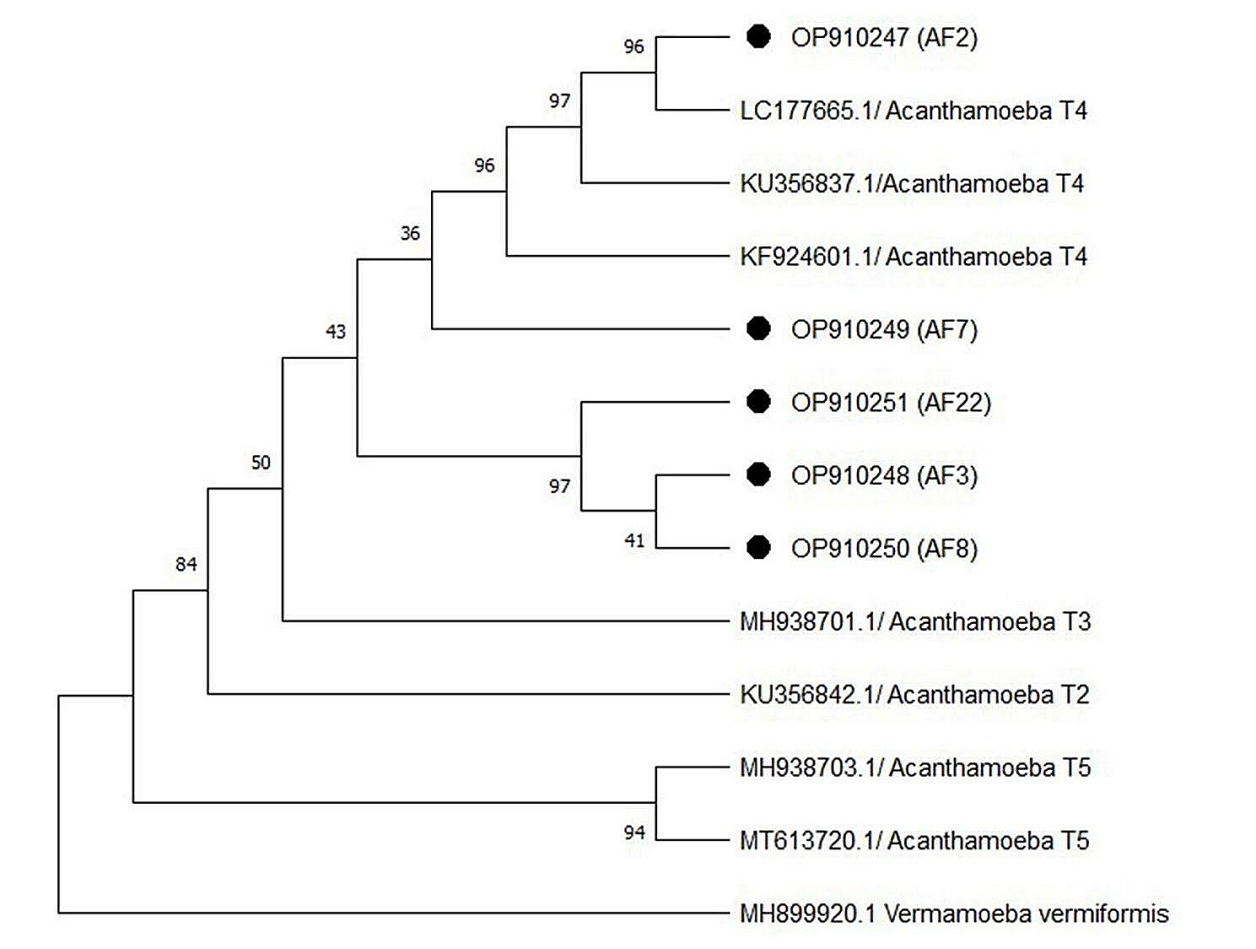
Taxonomic status of isolated *Acanthamoeba* sp. from fish source shown in a phylogenetic tree based on the multiple sequence alignment of 18 S rRNA gene (ASA1 amplimer)

The phylogenetic method was used to compare the differences between the isolates obtained from this study and genotypes 2 to 5. The phylogenetic tree demonstrated that all sequences had a relationship with the *Acanthamoeba* genotype T4 as a reference sequence based on the *SSU rRNA* gene nucleotide sequences (Fig. 6).

## Discussion

*Acanthamoeba* persists in freshwater and marine environments due to cyst resistance to dehydration, freezing temperatures, and commercial disinfecting agents such as bromine, chlorine, and ozone [39]. In addition to being opportunistic pathogens, *Acanthamoebas* may act as environmental vectors for a swarm of bacterial pathogens, including *Chlamydia*, *Legionella*, and *Burkholderia* [40–42] and maybe, could serve as vectors for pathogenic bacteria in fish [43].

According to published literature, these amphizoic facultative parasites are primarily transmitted horizontally through the water [44]. The degree and manner of the host-parasite relationship is highly variable depending on different factors. Therefore, although in some cases, there may be only a light systemic infection (particularly those occurring in nature), in other cases, fish can become heavily parasitized leading to mass mortalities [45]. There may be some substantial differences in biological characteristics of *Acanthamoeba* strains, so that for example *Acanthamoeba polyphaga* strain MC-1 can survive in different species of fish for at least 60 days, penetrate fish tissues, spread systemically to all organs, and cause pathological lesions. The failure of the A-5 and Lilly A-1 strains of *A. culbertsoni* to survive in fish, on the other hand, would suggest that fish may not be suitable hosts for this species of amebae [45]. The main causes of these free-living amoebae becoming parasites and influence NGD development are much less understood and remain unknown than in seawater AGD [13, 46]. However, there appears to be a link between this phenomenon and the exposure of fish to environmental stressful factors that impair the functionality of the immune system and elevate susceptibility of the fish against NGD. In addition, coinfection by different bacterial, fungal, and parasitic infectious agents complicates the diagnosis and prognosis [13, 30].

A combination of clinical and histopathological findings was recorded and molecular genetic analyses was used to determine the genus and genotype of free-living amoebae isolated from fish lesions. The appearance of multifocal patches of pale, swollen gill tissue and mucus hypersecretion on the gills and skin as well as lethargy, inappetence, and body discoloration following amoebic invasion in discus fish (*S. aequifasciatus*) was clinically very similar to other cases of NGD previously described in various fish species [25, 33, 44, 47].

The histopathological findings revealed that gill pathology was consistent with AGD and NGD based on some diagnostic criteria including lamellar epithelial hyperplasia, lamellar fusion and gill vesicles (interlamellar spaces or cavities). Spongiosis was also observed in the distal portion of the gill filaments of farmed brown trout (*Salmo trutta* L.) due to nodular gill disease [39]. In the current study all of these lesions were observed which is consistent with previous studies.

Presence of numerous cysts of *Acanthamoeba* sp. in gills of fish have not been mentioned in the previous reports of NGD in fish. However, these findings were reported in human cases. Numerous cysts of *Acanthamoeba* sp. in lung and some scattered cysts with double-layered walls in amoebic encephalitis have been described [48, 49]. In the present study similar cysts were observed in the gills.

Some amphizoic species may cause severe disease with a high mortality rate (e.g., intestinal amoebiasis, systemic amoebiasis, and AGD) [50]. Although amoeba identification in internal tissue sections can be difficult, some amoeba-specific features, such as cytoplasmic vacuoles, may be visible and the cytoplasm has a ‘foamy’ appearance due to the presence of prominent vacuoles [50]. In the present study, foamy appearance of trophozoites of *Acanthamoeba* sp. was observed in some cases. This finding confirmed that intestinal amoebiasis can be accrued in discus fish which is consistent with findings of Guz and Szczepaniak [47].

Molecular-based assays have become widely used for detecting aquatic pathogens due to their sensitivity, specificity, and reproducibility [51, 52]. Although there is still a limitation of pathogenic aquatic amoeba assays available [51], the amoeba assays that have been developed play an important role in answering a variety of etiological questions, such as determining host specificity, as well as agent identity, distribution, and abundance [53, 54].

With the growing global trade in aquatic products, the risk of disease transmission is undeniably a major concern. The vast literature on pathogenic free-living amoebae has dedicated special emphasis to human pathogens, including strains of the genus *Acanthamoeba*. In addition to *Acanthamoeba* spp., several amoeba species, including *Naegleria fowleri*, *Balamuthia mandrillaris*, *Entamoeba* complex, and *Sappinia* sp., are considered common human pathogens [55, 56], and may cause significant clinical complications such as granulomatous amoebic encephalitis (GAE), cutaneous acanthamoebiasis (CA), amoebic keratitis (AK), and primary amoebic meningoencephalitis (PAM) [57, 58] as well as corneal ulceration (CU) and blindness [45] in humans. Therefore, this disease is also important from a zoonotic standpoint, and further research will be required in the future, particularly in the field of transmission and how the causative agent is transmitted from fish to humans.

## Conclusion

The present study aimed to identify the presence of the Acanthamoeba T4 genotype on the skin and gills of discus fish exhibiting signs of illness in freshwater ornamental fish farms. This observation suggests the potential for a transmission of amoeba infection from ornamental fish to humans, thereby highlighting the need for further investigation into this infection among ornamental fish, as well as individuals who interact with them and their environment. To the authors’ knowledge, this study represents the first molecular investigation of acanthamoebiasis in discus fish (*S. aequifasciatus*).

## Data Availability

No datasets were generated or analysed during the current study.
